# Impacts of gold nanoparticle charge and ligand type on surface binding and toxicity to Gram-negative and Gram-positive bacteria†
†Electronic supplementary information (ESI) available: Representative transmission electron micrographs of AuNPs, sample images from colony counting assays, representative cytograms, calculation to determine PAH-ligand concentrations on PAH–AuNPs, and a video of dark field TEM images of *Bacillus* cells with PAH–AuNPs. See DOI: 10.1039/c5sc00792e



**DOI:** 10.1039/c5sc00792e

**Published:** 2015-06-16

**Authors:** Z. Vivian Feng, Ian L. Gunsolus, Tian A. Qiu, Katie R. Hurley, Lyle H. Nyberg, Hilena Frew, Kyle P. Johnson, Ariane M. Vartanian, Lisa M. Jacob, Samuel E. Lohse, Marco D. Torelli, Robert J. Hamers, Catherine J. Murphy, Christy L. Haynes

**Affiliations:** a Chemistry Department , Augsburg College , Minneapolis , MN 55454 , USA . Email: feng@augsburg.edu; b Department of Chemistry , University of Minnesota , Minneapolis , MN 55455 , USA . Email: chaynes@umn.edu; c Department of Chemistry , University of Illinois at Urbana-Champaign , Urbana , IL 61801 , USA; d Department of Chemistry , University of Wisconsin , Madison , WI 53706 , USA

## Abstract

Although nanomaterials facilitate significant technological advancement in our society, their potential impacts on the environment are yet to be fully understood. In this study, two environmentally relevant bacteria, *Shewanella oneidensis* and *Bacillus subtilis*, have been used as model organisms to elucidate the molecular interactions between these bacterial classes and Au nanoparticles (AuNPs) with well-controlled and well-characterized surface chemistries: anionic 3-mercaptopropionic acid (MPA), cationic 3-mercaptopropylamine (MPNH_2_), and the cationic polyelectrolyte poly(allylamine hydrochloride) (PAH). The data demonstrate that cationic, especially polyelectrolyte-wrapped AuNPs, were more toxic to both the Gram-negative and Gram-positive bacteria. The levels of toxicity observed were closely related to the percentage of cells with AuNPs associated with the cell surface as measured *in situ* using flow cytometry. The NP concentration-dependent binding profiles were drastically different for the two bacteria strains, suggesting the critical role of bacterial cell surface chemistry in determining nanoparticle association, and thereby, biological impact.

## Abstract 2

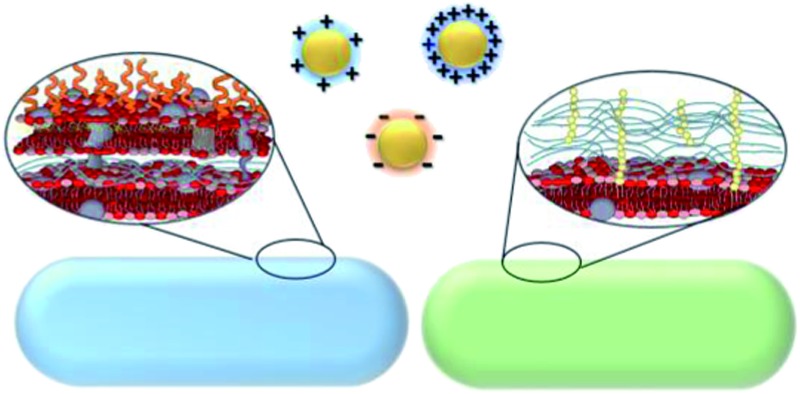
Higher cationic charge density on nanoparticles is correlated with higher toxicity to bacteria.

## Introduction

As the breadth of chemical and physical properties achieved within nanomaterials has expanded, so too has the number of nanomaterial-containing products. From antimicrobial clothing to high-efficiency catalytic converters in electric vehicles, engineered nanomaterials have greatly benefited our society.^1,2^ Inevitably, these materials are now introduced into the environment both intentionally and unintentionally. In recent years, significant research effort has been devoted to understanding engineered nanoparticle toxicity to mammalian cells in order to assess their potential impacts on human health.^3–5^ Equal attention to the environmental impacts of nanomaterials is required to ensure their short-term environmental safety and to prevent long term adverse effects, the remediation of which is likely to be more costly than preventative research.^1,6^

Bacteria play various vital roles in the ecosystem, including nutrient cycling and environmental remediation. At the bottom of the food chain, they also become an important entry point for nanomaterials to potentially interact with higher-trophic-level organisms.^7^ Accordingly, bacteria are excellent single cell model organisms to assess the environmental toxicity of engineered nanomaterials. Knowledge of their mechanisms of interaction with nanomaterials may also guide the redesign to more environmentally benign materials.

There are many challenges associated with studying the interactions between nanomaterials and bacteria. The field of microbial nanotoxicity assessment is populated with studies focused on the impacts of nanomaterials on bacterial growth and viability,^8–11^ often lacking molecular insight into the mechanism of toxicity. This is largely due to the paucity of effective methods to perform *in situ* examination of bacterial-nanoparticle interactions.

Herein, we investigate the surface association of well-characterized engineered gold nanoparticles (AuNPs) with two bacterial model species, using flow cytometry and transmission electron microscopy (TEM). In parallel, we assess the toxicity of these nanoparticles and relate their toxicity to the extent of cell–surface association. Although studies have often linked bactericidal properties of NPs with their affinities for cell surfaces,^9,12–14^ most of these investigations employed *ex situ* methods to characterize the interaction. The results presented herein demonstrate a powerful application of flow cytometry utilizing the optical properties of NPs to interrogate the complex nano-bio interface *in situ*, allowing correlation of NP-to-cell association and NP effect on cell viability. This work demonstrates that both bacterial cell surface chemistry and nanoparticle surface chemistry influence nanoparticle-bacterial interactions, hence impacting toxicity.

Bacteria, based on the structure of their cell walls, are categorized as either Gram-negative or Gram-positive. Because cell walls are often the point of contact to the external world, differences in cell wall structures may result in varied interactions between bacteria and nanomaterials. Gram-negative bacteria feature two lipid membranes, an outer and a cytoplasmic membrane, with a thin peptidoglycan layer in-between.^15^ The outer membrane is heavily populated with lipopolysaccharides (LPS), which have been suggested to protect bacteria from antibiotics.^16^ Cell surfaces are negatively charged due primarily to phosphate groups as well as carboxylate groups present in sugar acids. Gram-positive bacterial cell walls are composed of a thick peptidoglycan layer (15–100 nm)^15,17^ with polymeric teichoic acids, and a cytoplasmic membrane underneath. Cell surfaces are negatively charged, largely due to the teichoic acid polymeric chains which contain anionic phosphate groups in the glycerolphosphate repeat units.^15,18^ The teichoic acid chains, as well as the peptidoglycan layer, are essential for maintaining cellular integrity and have been suggested to be binding sites for divalent cations in solution.^15^ In this study, environmentally beneficial bacteria *Shewanella oneidensis* MR-1 (Gram-negative) and *Bacillus subtilis* (Gram-positive) were selected as model organisms.

**Fig. 1:**
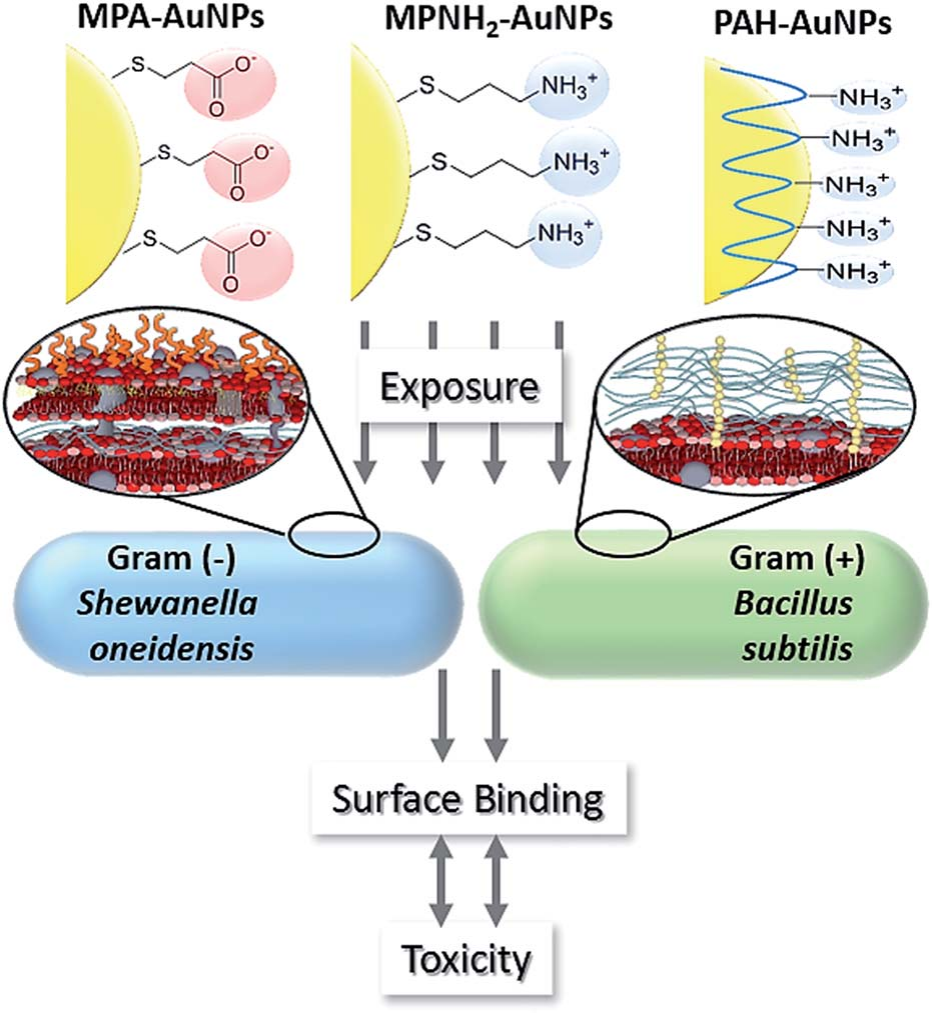
Schematic of overall experimental design.

This study employed three types of engineered AuNPs with different surface stabilizers: an anionic ligand, 3-mercaptopropionic acid (MPA); a cationic ligand, 3-mercaptopropylamine (MPNH_2_); and a cationic polyelectrolyte, poly(allylamine hydrochloride) (PAH). Both MPA and MPNH_2_ surface ligands covalently bond to AuNP surfaces, while PAH is a long-chain polymer that physically wraps around the AuNPs without covalent linkages (shown in Fig. 1). All three NPs have a gold core of sub-ten-nm-diameter. Au was chosen as the core material because of its chemical inertness, well-characterized plasmonic properties, and increasing applications in medical and consumer products.^19,20^

## Experimental

### Materials

All materials were used as received, unless otherwise noted. Hydrogen tetrachloroaurate trihydrate (HAuCl_4_·3H_2_O), poly(allylamine hydrochloride) (15 000 *M*_w_), 3-mercaptopropionoic acid, 3-aminopropane thiol hydrochloride (3-mercaptopropylamine), and sodium borohydride (NaBH_4_) were purchased from Sigma-Aldrich (St Louis, MO). Trisodium citrate dihydrate was purchased from Flinn Scientific (Batavia, IL). Pall tangential flow filtration capsules (50 kDa pore size) were purchased from VWR (Radnor, PA). Nanopure deionized water was prepared using a Barnstead Diamond Nanopure filtration system. All glassware used in nanoparticle synthesis was cleaned prior to use with *aqua regia*. SiO/Cu mesh and 200 mesh copper with carbon film and formvar support TEM grids were obtained from Ted Pella (Redding, CA).

### AuNP syntheses and characterizations

All three types of AuNPs used in this work were synthesized following existing protocols.^21–23^

#### MPA–AuNPs

400 mL of nanopure deionized water, 1.7 mL of HAuCl_4_ (0.1 M), 0.8 mL of NaOH (1.0 M), and 0.2 mL of mercaptopropionic acid (0.1 M) were stirred at vortex for 10 min. Then, 5.7 mL of fresh sodium borohydride solution (0.1 M) was added to the flask, and the solution rapidly turned red-brown. The AuNP solution was stirred for 3 h and purified through diafiltration, where 500 mL AuNP solution was concentrated to a volume of 25 mL and washed with 2.0 L of nanopure deionized water.

#### MPNH_2_–AuNPs

400 mL of nanopure deionized water, 0.9 mL of HAuCl_4_ (0.1 M), 5.7 mL of HCl (0.1 M), and 0.5 mL of mercaptopropylamine (0.1 M) were stirred at vortex for 10 min. Then, 4.0 mL of fresh sodium borohydride solution (0.1 M) was added to the mixture. The solution rapidly turned red-brown and was stirred for 3 h. The MPNH_2_–AuNPs were then purified by diafiltration, as described above.

#### PAH–AuNPs

PAH-functionalized AuNPs were synthesized by polyelectrolyte wrapping of 4 nm citrate-AuNPs according to previously reported procedures.^23–26^ In a typical flow reactor synthesis, 20.0 mL of HAuCl_4_ (0.01 M) and 6.0 mL of sodium citrate (0.1 M) were combined in an 2 L Erlenmeyer flask containing 1600 mL of nanopure deionized water. In a second 2 L Erlenmeyer flask, 1614.0 mL of nanopure deionized water was chilled in an ice-water bath. 12.0 mL of chilled NaBH_4_ (0.1 M) was added to the cold flask, which was swirled briefly. A flow line was placed into each 2 L flask and the flow reactor pump was started at a setting of 40 mL min^–1^. Once the two solutions combined in the flow reactor line, the solution turned a light red-brown, and the synthesized particles were collected in a 4 L polyethylene bottle with gentle stirring. The resulting citrate-AuNP solution was then stirred for at least 3 h. The 4 nm citrate-AuNPs were then wrapped with 15 000 *M*_w_ PAH to prepare 4 nm PAH–AuNPs, as previously described.^25^ To the approximately 3.2 L of as-synthesized particles, 32.0 mL of NaCl (0.1 M) and 100.0 mL of a PAH solution (10 mg mL^–1^ in 0.001 M NaCl) were added with vigorous stirring. The nanoparticles were then allowed to stir overnight in the wrapping solution. The PAH–AuNPs were subsequently purified by centrifugation and washing (13 000 × *g* for 55 min).

All three AuNP types were characterized using a combination of transmission electron microscopy (TEM), UV-vis extinction spectroscopy, *ζ*-potential analysis, and dynamic light scattering (DLS). UV-vis extinction spectroscopy analysis of the localized surface plasmon resonance (LSPR) was performed using a Cary 500 Scan UV-vis-NIR Spectrophotometer. For TEM analysis, a small volume of the relevant purified AuNP solution was dropcast onto a TEM grid, and the AuNPs were imaged using a JEOL 2100 TEM. TEM images were then analyzed using ImageJ software to determine the size distribution of the AuNPs with a minimum of 250 nanoparticles measured in each condition. DLS and *ζ*-potential (Brookhaven ZetaPALS) were used to determine aggregate sizes and stability of the AuNPs in nanopure deionized water and bacterial media prior to further experiments.

For X-ray photoelectron spectroscopy, particles were centrifuged at 14 100 × *g* until pelleted and resuspended in minimal nanopure water to remove excess ligands. Particles were then dropcast onto conductive silicon (P doped, <0.004 Ω cm) and dried at thickness sufficient to fully attenuate the substrate signal. XPS spectra were obtained in a custom-built, ultrahigh-vacuum Phi XPS system with a base pressure of <2 × 10^–10^ Torr. X-rays were produced by an Al Kα source with a quartz-crystal monochromator. Typical measurements used pass energies of 46 eV (yielding analyzer resolution of 0.64 eV). An electron collection angle of 45° with respect to the surface normal was used for all measurements.

### Bacterial culture and AuNP exposure

Shewanella oneidensis MR-1 stock was a gift from the lab of Jeff Gralnick at the University of Minnesota. *Bacillus subtilis* strain SB 491 was purchased from *Bacillus* Genetic Stock Center (Columbus, OH). Bacteria liquid cultures were grown in Luria Broth media overnight at 30 °C to late-log phase from colony inoculants on solid agar plates. Cells were harvested by centrifugation for 10 min at 750 × *g*, washed in Dulbecco's phosphate-buffered saline (D-PBS) buffer, and exchanged into a HEPES buffer (2 mM HEPES and 25 mM NaCl, at pH 7.4). The cultures were then diluted to OD 0.2 at 600 nm (OD_600_) to achieve a cell density of approximately 2 × 10^8^ cells per mL and then incubated with AuNP solutions for 10 minutes before association/toxicity analyses.

### Bacterial toxicity assays

#### Respirometry

Cell suspensions were grown in aqueous media (buffered with 10 mM HEPES and containing 11.6 mM NaCl, 4.0 mM KCl, 1.4 mM MgCl_2_·6H_2_O, 2.8 mM Na_2_SO_4_, 2.8 mM NH_4_Cl, 0.088 mM Na_2_HPO_4_, 0.051 mM CaCl_2_, and 100 mM sodium lactate for *Shewanella* or 10 mM dextrose for *Bacillus*) over 24 hours. The cell density was then adjusted to 2 × 10^8^ cells per mL, and this suspension was diluted 1 : 10 into fresh media. One hundred milliliter aliquots of this diluted cell suspension were placed into 125 mL glass vessels containing removable rubber septa, and aliquots of concentrated nanoparticle solutions were added to achieve the desired exposure concentration. Inserts containing concentrated KOH (aq.) were placed into the headspace above the culture, and the vessels were subsequently sealed. Vessels were placed into a water bath maintained at 30 °C for *Shewanella* and 37 °C for *Bacillus*, and the suspensions were stirred continuously at 500 rpm. A small gauge needle was placed through each septum, and tubing (Tygon® 4040-A) linked each vessel to a respirometer system (Respirometer Systems and Applications, Inc., Springdale, AK) that monitored cellular consumption of O_2(g)_ over 48 h. As the cell population size increased over time, total aerobic respiratory activity also increased. Aerobic respiration consumes O_2(g)_ and produces CO_2(g)_. The latter is removed from the gas phase by reaction with concentrated KOH_(aq.)_. Cellular respiration thus decreased the total pressure in the sealed vessels, and O_2(g)_ was supplied as needed at 10 minute intervals to maintain a constant pressure. The total mass of O_2(g)_ delivered to each vessel was recorded at 10 minute intervals over 48 h.

#### Colony counting assays

The colony counting method was used to examine the concentration-dependent toxic effect of the cationic AuNPs on both bacterial strains. Following the cell preparation steps described above, cell suspension in HEPES buffer at OD ∼0.2 was diluted to about 10^4^ colony-forming units (CFUs) per mL in HEPES buffer. These cells were treated with various concentrations of AuNPs or free ligand solutions and incubated for 10 minutes. The drop plate method was used for *Shewanella* by adapting a previously described method.^27^ Briefly, after sufficient mixing, 10 μL of control or treated bacterial culture was dotted onto the surface of 1.5% LB agar plates that were pre-treated by drying in 30–32 °C oven and UV-illuminating for 15 minutes for sterilization. After drops were completely absorbed in the agar, plates were incubated upside down at 30 °C for 24 hours before colonies were counted using a Bantex Colony Counter 920A. The viability of cells from each treatment was reported as a ratio to its control samples.

Due to the swarming mobility of *Bacillus subtilis*,^28^ the pour plate method of colony counting was used instead. In this method, 60 μL of AuNP-incubated *Bacillus* cell suspension and 1 mL of melted LB-agar solution at ∼45 °C (1.5% agar) were poured and mixed in each well of a 12-well plate. The plates were incubated at 37 °C for 18–20 hours, and the colonies in each well were counted. The viability of cells from each treatment was reported as a ratio to its control samples.

### Characterization of NP-bacteria interactions

#### Flow cytometry

AuNP-incubated bacterial suspensions at 2 × 10^8^ cells per mL were mixed 1 : 1 with 3.34 mM SYTO 9 (Life Technologies Kit L7012), a nucleic acid stain. Following a 15 min incubation at room temperature, nanoparticle association with bacterial cells was analyzed using a Becton Dickenson LSRII SORP flow cytometer equipped with a 20 mW, 488 nm laser. SYTO 9 fluorescence intensity was monitored to discriminate cells from debris present in solution, and orthogonal (side) light scattering intensity based on the plasmonic extinction of the Au nanoparticles was monitored to identify cell-bound nanoparticles. A total of 30 000 cells were analyzed from each condition, and the subpopulation of bacterial cells associated with nanoparticles was counted.

#### TEM analysis

Biological TEM samples were prepared by a typical process of fixation, dehydration, and embedding in a resin matrix.^29,30^ Briefly, bacterial suspensions in HEPES at OD 0.2 were pelleted and washed three times in 0.1 M sodium cacodylate buffer, then fixed in a 2.5% gluteraldehyde in 0.1 M sodium cacodylate buffer solution for 1 hour. The pellet was flipped halfway through fixation to improve gluteraldehyde penetration. The pellets were washed in sodium cacodylate buffer again and then dehydrated in a series of graded ethanol solutions (30, 50, 70, 80, 90, 95, and 100% ethanol in water). The pellet was rinsed three times with propylene oxide (3 min each), then incubated in 2 : 1 propylene oxide : resin for 2 hours, 1 : 1 propylene oxide : resin overnight, and a fresh batch of 1 : 1 propylene oxide : resin for 8 hours. The pellets were then allowed to sit overnight in 100% resin. Finally, a new batch of resin was added, and the sample was cured at 40 °C for one day and then 60 °C for two days. Next, 60–70 nm-thick samples were sliced off the resin block using a Leica EM UC6 Ultramicrotome equipped with a diamond knife, stained with uranyl acetate and lead citrate for enhanced contrast, and placed on 200 mesh copper grids with carbon and formvar supports (Ted Pella Inc.) for imaging.

All room temperature TEM images were collected on a Tecnai T12 transmission electron microscope operating at 120 kV. Dark field TEM images were collected in dark field mode with a variety of objective aperture sizes depending on the instrument magnification.^30^

## Results and discussion

### AuNP characterization

AuNPs were characterized with a variety of methods; this in-depth characterization is critical for optimal interpretation of nanoparticle/cell interaction. Table 1 summarizes the size and surface chemistry characteristics of the three AuNP preparations considered herein. Representative TEM images of these NPs are provided in the ESI (Fig. S1†). Overall, TEM images showed that both MPA– and PAH–AuNPs were similar in size (∼4.5 nm-diameter) and polydispersity (±∼1 nm), while MPNH_2_–AuNPs are larger (8.9 nm) and more polydisperse. Dynamic light scattering experiments to evaluate the hydrodynamic diameters of the NPs either in water or HEPES buffer (used for biological exposures) were attempted, but the small nanoparticle sizes were below the limit of detection of the DLS instrument; this indicates that the nanoparticles were not aggregating to a significant extent. *ζ*-potentials of the three nanoparticles did not change significantly after transferring particles from water to HEPES buffer. These results indicate that the buffer used for biological exposures had minimal impact on NP surface charge.

**Table 1:** Characterization Results for AuNPs

AuNP	MPA–	MPNH_2_–	PAH–
LSPR *λ*_max_ (nm)	512	521	524
*d*_core_ ^*a*^ (nm)	4.2 ± 1.2	8.9 ± 3.0	4.7 ± 1.5
*ζ*-potential (mV) (in H_2_O)	–36.0 ± 1.4	26.7 ± 6.7	38.4 ± 1.8
*ζ*-potential (mV) (in HEPES)	–37.5 ± 3.9	26.9 ± 2.5	35.1 ± 3.4
Charge density^*b*^ (charge per nm^2^)	5.6 (5.2–6.0)	4.6 (4.1–4.9)	12.8 (11.2–14.1)

^*a*^Based on TEM image analysis (*n* ≥ 250 AuNPs counted).

^*b*^Ranges are provided instead of a standard deviation due to the asymmetry that arises in error propagated by a varying radius.

Charge density of AuNPs was measured using XPS, also shown in Table 1. Charge densities correlate directly to ligand densities, which are determined by measurement of ligand shell and nanoparticle core, in this case C (1s), N (1s), S (2p), and Au (4f) electrons. Because nanoparticle size is known, the expected ratios can be predicted computationally and compared to experimental values to derive a ligand density.^21,31^ The results indicated that MPA– and MPNH_2_–AuNPs had comparable ligand coverage, while PAH–AuNPs had a significantly higher surface charge density.

### Bacterial viability upon NP-exposure

**Fig. 2:**
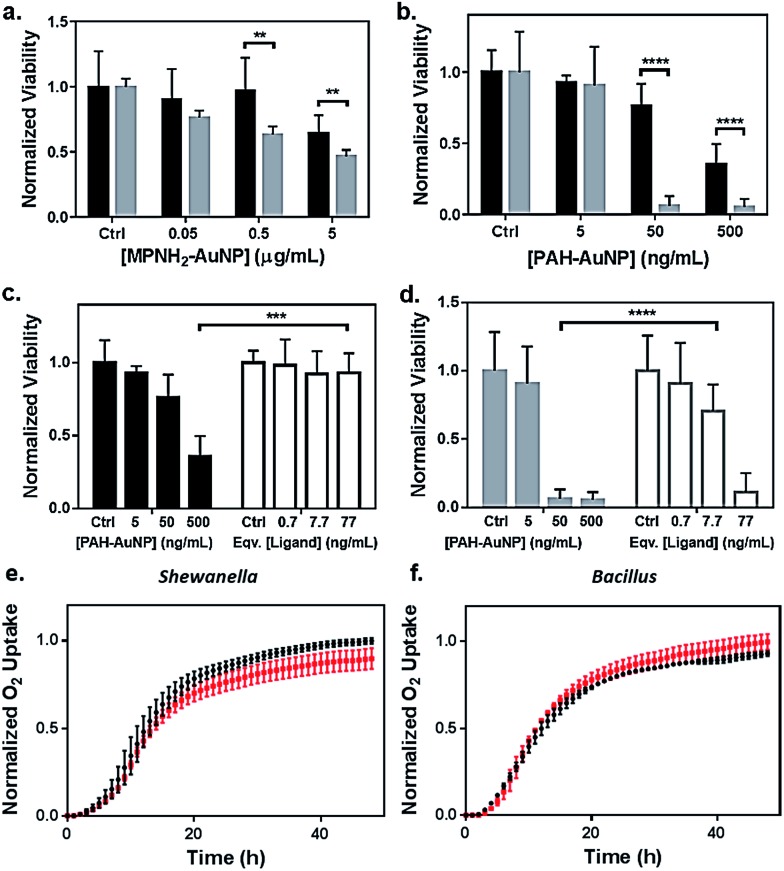
Bacterial viability assessed by colony counting methods (a–d) and respirometry (e and f). Dose-dependent toxicity assessment of (a) MPNH_2_–AuNPs and (b) PAH–AuNPs on *Shewanella* (black bars) and *Bacillus* (grey bars), and comparisons of toxicity between PAH–AuNPs and PAH free ligand on *Shewanella* (c) and *Bacillus* (d). Ligand concentrations were chosen based on XPS measurements of ligand coverage on nanoparticle surfaces.^31^ Representative respirometry analysis of (e) *Shewanella* and (f) *Bacillus* without (black circles) and with (red squares) 5 μg mL^–1^ MPA–AuNPs in growth media. The O_2_ consumption curve can be interpreted similarly to growth curves obtained through optical density measurements to assess the impact of NPs on the bacterial strains.

Toxicity of the AuNPs to both bacteria models was assessed using respirometry, which monitors O_2_ consumption to reflect bacterial viability and population growth. Results showed that exposure to 5 μg Au/mL anionic MPA–AuNPs had minimal toxic effect on either *Shewanella* or *Bacillus* (Fig. 2(e) and (f)), while both cationic AuNPs impacted the growth of both bacterial species to different extents. This observation was in agreement with earlier studies comparing the toxicity of cationic *vs.* anionic nanoparticles on various bacterial models.^13,32^

To investigate the nanoparticle concentration-dependent response of both bacterial species to cationic AuNPs (*i.e.*, those functionalized with MPNH_2_ and PAH), colony counting methods were used. Liquid suspensions of bacterial cells were exposed to NPs and subsequently plated onto nutrient-rich agar plates. Distinct bacterial colonies formed over 24 h. Reductions in colonies upon exposure to toxic materials served as a metric of toxicity. Due to the differences in bacterial colony morphology, the drop plate method was employed for *Shewanella* and the pour plate method was used for *Bacillus* (representative appearances of resulting colonies are shown in Fig. S2†).

Fig. 2(a) compares the toxicity of MPNH_2_–AuNPs to both *Shewanella* and *Bacillus* at concentrations ranging from 0.05 to 5 μg Au/mL. These results showed that the MPNH_2_–AuNPs were not toxic to *Shewanella* at doses lower than 5 μg mL^–1^ (unpaired *t*-test, *p* < 0.0001), while a minor reduction in the viability of *Bacillus* was observed following exposure to 0.05 μg Au/mL (unpaired *t*-test, *p* < 0.01). Comparing the toxicity of PAH–AuNPs towards *Bacillus vs. Shewanella* at concentrations from 5 to 500 ng mL^–1^, it is clear that *Bacillus* is more prone than *Shewanella* to negative impacts from the nanoparticles, as depicted in Fig. 2(b). Following exposure to 50 ng mL^–1^ PAH–AuNPs, less than 10% of *Bacillus* cells formed colonies, while 80% of *Shewanella* were still viable. Following exposure to 5 μg mL^–1^ PAH–AuNPs, no colonies were formed in *Bacillus*, indicating a highly toxic effect of the PAH–AuNP suspension at this dose.

To control for the possible contribution of unbound nanoparticle surface ligand to the observed toxicity of PAH–AuNPs, viability studies were performed comparing the effects of PAH–AuNPs with PAH ligands on both bacteria, shown in Fig. 2(c) and (d). The concentrations of PAH ligands used were estimated based on XPS measurements of PAH–AuNP ligand density on the ∼4 nm-diameter AuNPs with the assumption that no unbound free PAH ligand was present in the AuNP solution during XPS analysis (see calculation in ESI†). The calculation indicated 77 ng mL^–1^ of PAH was present on the surface of 500 ng mL^–1^ of PAH–AuNPs. Hence the ligand amounts are denoted as “equivalent [ligand]” in Fig. 2(c) and (d). Overall, both Fig. 2(c) and (d) show that this concentration of ligands alone does not account for the toxicity measured when the ligands were presented on AuNPs. For *Shewanella*, 500 ng mL^–1^ AuNP resulted in >50% colony reduction, while the corresponding amount of ligand (77 ng mL^–1^) was not toxic to the cells (unpaired *t*-test, *p* < 0.001). Similarly, at an exposure concentration of 50 ng Au/mL, AuNPs were highly toxic to *Bacillus*, while the corresponding 7.7 ng mL^–1^ PAH free ligand was significantly less toxic (unpaired *t*-test, *p* < 0.0001). We note that enhanced toxicity of a charged ligands presented on NP surfaces *vs.* in solution has been reported in the multi-cell model organism, *Daphnia magna*,^21^ and we hypothesize that differences in toxicity observed here are due to a higher localized surface charge when PAH is presented to cell surfaces on AuNPs *vs.* as free polymeric chains in solution.

Although both MPNH_2_– and PAH–AuNPs are positively charged, the former are much less toxic than the latter to both bacterial species studied. The differences in toxicity could be attributed to the NP *ζ*-potentials and charge densities, as shown in Table 1. More positively charged NP surfaces and higher charge densities may yield stronger electrostatic interactions between PAH–AuNP and the negatively charged bacterial surfaces.

Comparing *Bacillus* with *Shewanella*, it is also clear that both MPNH_2_– and PAH–AuNPs are significantly more toxic to *Bacillus* than *Shewanella*. Other studies have pointed to the differences in toxic responses between model Gram-negative (*e.g. E. coli*, *P. aeruginosa*) and Gram-positive (*e.g. B. subtilis*, *S. aureus*) bacteria to various nanoparticles, many of which have observed a notably higher toxicity of nanoparticles to Gram-positive bacteria than that measured in Gram-negative ones.^14,33–35^ For Gram-negative bacteria, the lipopolysaccharide (LPS) structure has been identified as a protective layer controlling the surface interactions between bacteria and other species in the media.^16,34,36^ The differences in bacterial cell wall structures, *i.e.* the lack of an outer membrane with LPS, is likely the source of the more intense adverse effects observed for Gram-positive bacteria.

### Flow cytometry analysis of cell–NP binding

Flow cytometry is a powerful tool to rapidly screen and sort large volumes of cells. In bacterial studies, it has been often used in conjunction with fluorescence dyes to determine the viability of bacterial cultures.^37–39^ Herein, flow cytometry was performed as a high throughput method to quantitatively investigate the extent of AuNP association with the bacterial cell surface in order to correlate nanoparticle association with induced toxicity. Using this method, ten thousand cells were screened individually *in situ* to identify the presence of AuNPs on the cell surfaces in a matter of seconds. A membrane permeant nucleic acid-binding fluorescent dye, SYTO 9 (*λ*_ex_ = 488 nm, *λ*_em_ = 520 nm) was used to distinguish whole cells from cellular debris which lack nucleic acid content. When unstained and intact bacterial cells pass through the flow cytometer's interrogating laser beam without associated AuNPs, low signal intensity was observed in both the SYTO 9 detector channel (530 ± 10 nm) and the side (orthogonal) scattering channel (Fig. S3(a)†). When cells are incubated with SYTO 9 dye, the cell population shifts significantly along the horizontal axis to higher fluorescence intensity values (Fig. S3(b)†). Due to the high side scattering signal generated by AuNPs based on their LSPR, cells bound to AuNPs display significantly higher side scattering signal, resulting in a noticeable shift to higher values on the *y*-axis (Fig. S3(c)†). Thresholds on both side scattered light intensity and SYTO 9 fluorescence intensity were set using control samples exposed to either just SYTO 9 or AuNPs. Hence, the population of cells that is positive for both SYTO 9 and AuNPs (blue population in Fig. S3(d)†) can be quantified, and the size of this population relative to the overall cell population stained with SYTO 9 gives the fraction of intact cells associated with AuNPs.

**Fig. 3:**
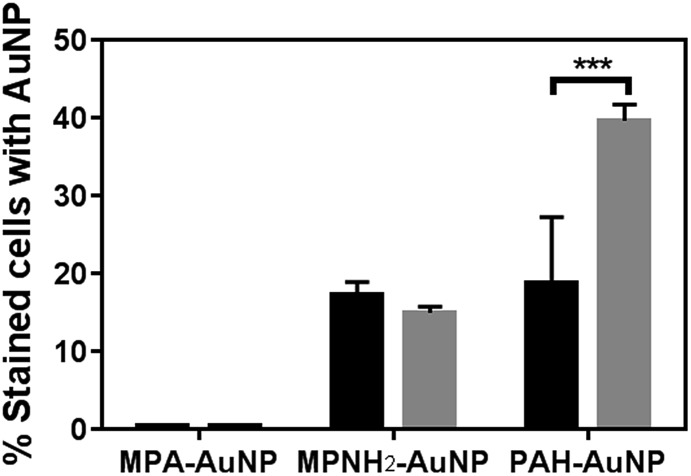
Flow cytometry-based bacteria-NP association comparison of *Shewanella* (black bars) and *Bacillus* (gray bars). All AuNPs were presented at the 5 μg mL^–1^ level (*** represents *p* < 0.001).

Fig. 3 summarizes the flow cytometry results. The populations of both *Shewanella* and *Bacillus* that have MPA–AuNPs on the surface are nearly negligible. In contrast, both positively charged AuNPs associated significantly with both types of cells. These observations are consistent with expectations based on the surface charges of these AuNPs, which are attracted to the negatively charged cell surfaces. Although the percentages of each bacterial cell species associated with MPNH_2_–AuNPs are not statistically different, the population of *Bacillus* with associated PAH–AuNPs is significantly greater than that of *Shewanella* (unpaired *t*-test, *p* < 0.001).

Our results from flow cytometry and bacterial viability studies suggest a correlation between the number of cells associated with AuNPs and corresponding NP toxicity to the organisms. To summarize these results: exposure to MPA–AuNPs, which associated minimally with either of the bacterial strains, resulted in the lowest toxicity of the AuNP types studies while PAH–AuNPs, which associated significantly with both *Shewanella* and *Bacillus* population, induced the highest cell death.

In considering the molecular interaction between the nanoparticles and bacteria, a variety of applications of cationic polyelectrolytes, either immobilized on substrates as antimicrobial materials^40,41^ or as colloidal particles as novel antibacterial drugs to combat multi-drug-resistant microbes, has emerged in the literature.^42–44^ The proposed bactericidal mechanism in these studies is through disruption of the integrity of cell membranes, leading to cell death. More relevant to our NP system, PAH has been identified to bind to phosphates.^44^ Abundant phosphate moieties are present in the teichoic acid chains on *Bacillus* cell surfaces and in the LPS layer of *Shewanella*, and this may explain the high PAH–AuNP surface association observed in both bacteria.

**Fig. 4:**
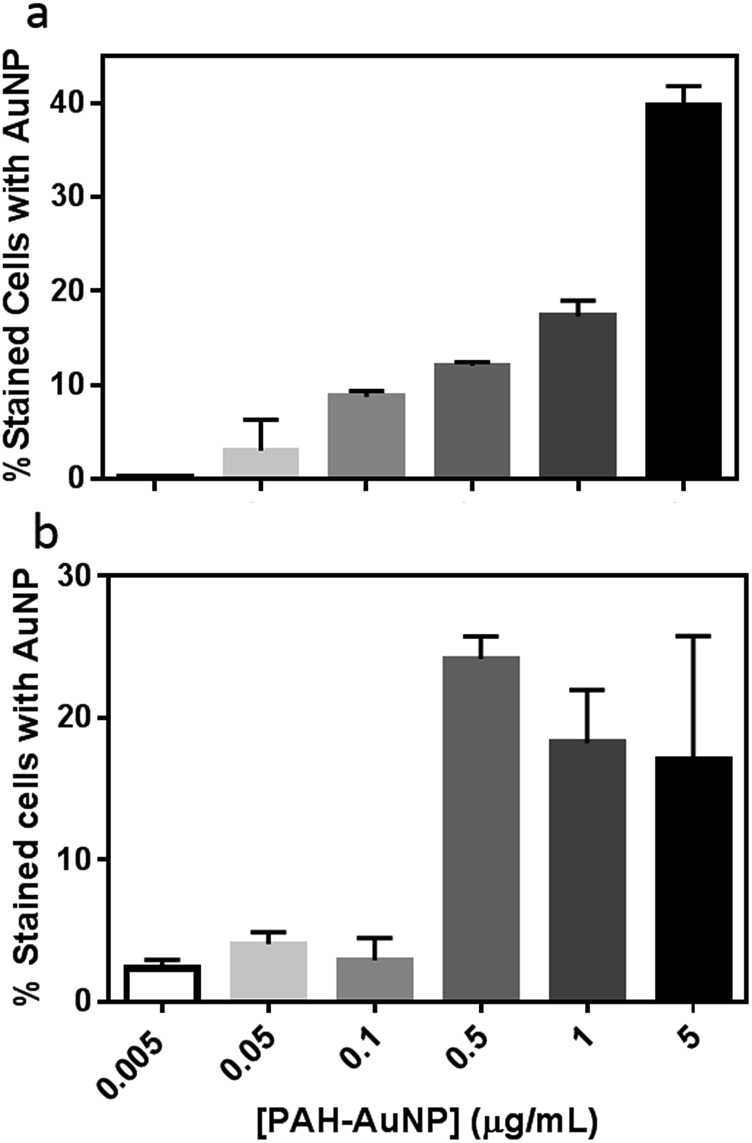
Concentration-dependence of PAH–AuNPs binding to (a) *Bacillus* and (b) *Shewanella*.

To further evaluate the PAH–AuNP interactions with both types of cells, the concentration-dependent association of the NPs to both cell populations was investigated. Fig. 4(a) demonstrates a clear concentration-dependent manner of PAH–AuNP associating with the *Bacillus* cells. To establish a rough estimate of NP affinity for the bacterial cells, a fit of the Langmuir adsorption isotherm model to our data provided a binding constant, *K*_b_, of 1.1 × 10^10^ M^–1^ for this interaction. This value is comparable with the binding constant reported by Boulos, *et.al.* ^45^ between 20 nm-diameter PAH–AuNPs and a model protein, bovine serum albumin (1.71 × 10^10^ M^–1^) using a fluorescence quenching titration method.

Interestingly, linear concentration-dependent binding was not observed between *Shewanella* and PAH–AuNPs, as shown in Fig. 4(b). Instead, the concentration-dependent association occurs in a step-wise manner. Below 0.10 μg mL^–1^ of AuNPs, the population of bacterial cells with AuNP association was minimal and independent of AuNP concentration. Above a concentration of 0.50 μg Au/mL, there is a sharp increase in the number of bacterial cells with AuNPs attached, yet increasing AuNP dose did not further increase this population.

Fig. 4 provides insights into various aspects of the bacteria–NP interactions. First of all, correlating concentration-dependent flow cytometry results with cell viability revealed that a similar degree of NP association may lead to different levels of toxicity in the Gram-negative *versus* Gram-positive bacterial strains. More specifically, at the 50 ng mL^–1^ level, both organisms have <10% of cells with AuNP on surfaces, yet less than 20% of *Bacillus* remain viable *vs.* 80% for *Shewanella*. This observation implies that the mechanism of PAH–AuNP toxicity is likely different between the two strains.

Secondly, the concentration-dependent binding profiles of the two bacteria are clearly distinct. The *Bacillus*/PAH–AuNP system exhibited an equilibrium relationship between adsorbate (PAH–AuNP) and adsorbent (cell surface), while the *Shewanella*/PAH–AuNP system revealed the presence of a critical energy barrier for attachment to occur. Papo, *et.al.* have proposed a mechanism of interaction between antimicrobial peptides and LPS from Gram-negative bacteria where electrostatic interactions resulted in surface accumulation of peptides until a threshold concentration of peptide was reached which led to LPS micellization and peptide entering to the lipid core region.^16^ It is likely that PAH presented on the AuNP surfaces may interact with LPS in a similar manner as peptides due to their polyelectrolytic nature. In this scenario, the 0.5 μg mL^–1^ PAH–AuNP may indicate the threshold level of surface density of PAH ligand present at *Shewanella* cell surface that led to micellization of LPS, granting the PAH–AuNPs access to the membrane bilayer.

### Transmission electron microscopy

**Fig. 5:**
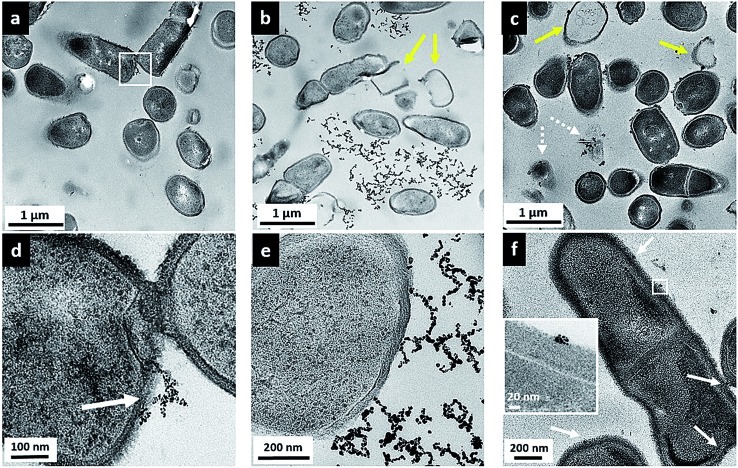
Transmission electron micrographs of *Bacillus* incubated with 5 μg mL^–1^ MPA–AuNP (a and d), 5 μg mL^–1^ MPNH_2_–AuNP (b and e), and 0.5 μg mL^–1^ PAH–AuNP (c and f). White arrows point to binding sites of NPs with cells; yellow arrows denote lysed cells or empty cells.

While the flow cytometry studies are quantitative and allow analysis of a large number of bacterial cells, these data give no information about how or where the nanoparticles are in relation to the bacterial cells. To visualize the surface interactions between these nanoparticles and the two bacterial models, sectioned TEM images were acquired to complement the flow cytometry data. Fig. 5 shows a series of TEM images of *Bacillus* in the presence of 5 μg mL^–1^ MPA–AuNPs (a and d), 5 μg mL^–1^ MPNH_2_–AuNPs (b and e), and 0.5 μg mL^–1^ PAH–AuNPs (c and f). A lower concentration was chosen for the PAH–AuNP because of the higher toxicity of this NP formulation observed. Panels (a), (b), and (c) at a lower magnification show the overall morphology of cells and nanoparticles, while (d), (e), and (f) at higher magnification reveal more specific interactions between cells and nanoparticles. The dark spots inside cells are exclusively stained ribosome structures, as often seen in *Bacillus* control samples (not shown), and similar to those reported earlier.^46^ Dark field TEM was also performed on these samples to distinguish AuNPs from any non-crystalline stain artifacts. Taking advantage of the highly crystalline structure of AuNPs, when imaged in this mode, NPs produced bright diffraction signal at various beam angles, allowing differentiation of crystalline AuNPs from amorphous stained cellular structures (see movie file in ESI† for an example). Overall, for all three nanoparticles, no internalization of AuNPs was observed into bacterial cells.

Fig. 5(a) shows that upon MPA–AuNP exposure, the majority of *Bacillu*s cells remain intact, and the extent of nanoparticle association is minimal. The lack of association was also seen macroscopically during TEM sample preparation where the bacteria-NP sample was pelleted after the 10 min incubation period. After multiple steps of buffer rinse, the majority of the MPA–AuNPs that were not cell-bound were washed off in the supernatant, leaving the *Bacillus* pellet white prior to embedding and sectioning. This observation is also in agreement with the low cytotoxicity and low surface association measured in flow cytometry for MPA–AuNPs and *Bacillus*. A magnified area (white square in Fig. 5(a)), shown in Fig. 5(d), demonstrates that where cell surface binding occurred, a small cluster of MPA–AuNPs was partially attached to the cell surface at various points, without compromising the integrity of the cell. In contrast, MPNH_2_–AuNPs induced cell lysis to a greater extent, as seen in Fig. 5(b), indicated by yellow arrows. Although some MPNH_2_–AuNPs are attached to cell surfaces at various points, a majority of the visible nanoparticles formed chain-shaped aggregates, similar to what was observed in TEM images of these NPs alone (Fig. S1(b)†), without a strong affinity for the cell surface (Fig. 5(e)). Lastly, PAH–AuNPs also induced cell lysis to a high degree, as shown in Fig. 5(c). More distinctively, nearly all nanoparticles in small clusters were bound to cellular species, whether it was intact cells, empty cell walls (yellow arrows), or cell wall-free cytoplasmic content (broken arrows). At a higher magnification, where *Bacillus* cell wall structure was resolved (inset in Fig. 5(f)), it is clear that NPs in small aggregates were attached to the thick peptidoglycan layer of the cell wall, far from the buried lipid membrane layer.

TEM studies of these AuNPs with *Bacillus* provided a snapshot of localized interactions. Although we refrain from analyzing these images quantitatively due to the highly localized and limited views presented, we note the correlation between the extent of cell lysis and the viability of *Bacillus* upon exposure to these NPs. The intermittent cell surface attachment of MPNH_2_–AuNPs induced some membrane deformation and lysis, similar to that shown in sectioned TEM of Gram-positive, *S. aureus* upon exposure to AuNPs with cationic surface ligands.^12^ TEM images also revealed that PAH–AuNPs, which showed the highest toxicity to *Bacillus*, had the highest affinity towards the cells and induced qualitatively severe cell lysis. Indeed, cationic polypeptides and polyelectrolytes have emerged as a new class of antibacterial drugs against multi-drug resistant strains of pathogens.^42,43,47^ It is highly likely that the high toxicity and strong affinity of PAH–AuNPs to bacteria cell features are largely due to the cationic polyelectrolyte coating on these particles. It is also clear that small clusters of PAH–AuNPs form upon attachment on cell surfaces. In the literature, a TEM study examining non-sliced bacteria interactions with 6 nm-diameter cationic AuNPs has reported AuNP clusters on the *Bacillus* surface that could be dispersed upon removal of surface proteins by trypsin.^48^ This evidence again may guide future studies of the molecular-level identification of cellular component(s) responsible for NP interactions.

**Fig. 6:**
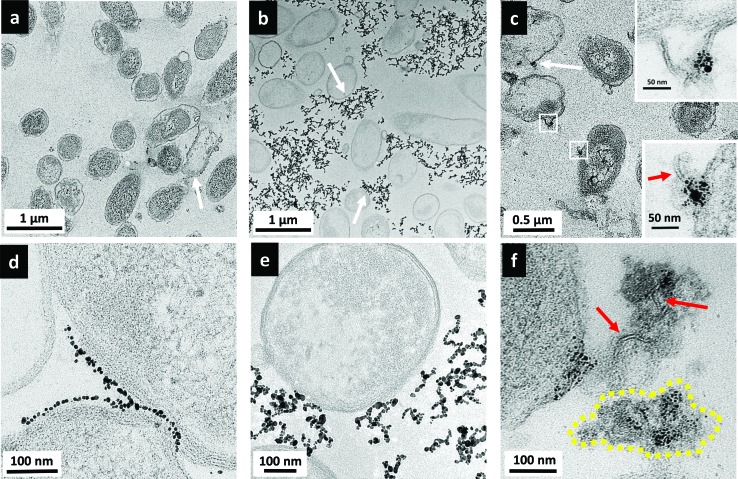
Transmission electron micrographs of *Shewanella* incubated with 5 μg mL^–1^ MPA–AuNP (a and d), 5 μg mL^–1^ MPNH_2_–AuNP (b and e), and 0.5 μg mL^–1^ PAH–AuNP (c and f). White arrows point to binding sites of NPs with cells; red arrows denote lipid bilayer-structure; yellow dashed-line indicates cytoplasmic content with multiple AuNPs attached.

Fig. 6 shows the parallel TEM images of *Shewanella* cells when exposed to the various AuNPs. *Shewanella*, like many other Gram-negative bacteria species, produces outer membrane vesicles (OMV).^49–51^ Such features were often observed in TEM images, but could not be attributed to the presence of AuNPs, based on comparisons with control samples not exposed to nanoparticles. Again, no internalization of NPs was observed.

Fig. 6(a) and (d) show the interactions between MPA–AuNPs with *Shewanella*. Overall, *Shewanella* cells remain intact. However, surprisingly, a few cells were uniformly packed with a thin layer of MPA–AuNPs on the bacterial periphery, contrary to the original hypothesis for this work based on electrostatic interactions. Again, macroscopically, during the TEM sample pelleting step, the cell pellet was stained dark purple after centrifugation, suggesting that some MPA–AuNPs remained with the cell pellet, which was different from the *Bacillus*/MPA–AuNP system. At higher magnification, as shown in Fig. 6(d), an interesting AuNP–cell interaction pattern was observed. MPA–AuNPs remain well separated, neatly lining the cell surfaces, yet keeping a small gap between the nanoparticles and the cell wall. The overall morphology of MPNH_2_–AuNPs with *Shewanella* resembled that of with *Bacillus* more closely. Chain-shaped NP aggregates are scattered around the cells with partial associations to cell surfaces, as shown in Fig. 6(b) and (e). A qualitative majority of the cells remain intact even when NPs are attached.

Initial examination of results from TEM and flow cytometry for MPA–AuNP binding on *Shewanella* may seem contradictory. Closer examination reveals that although a significant number of MPA–AuNPs were attached to some *Shewanella*, as shown in Fig. 6(d), the number of cells with AuNPs attached in this fashion remains small in the population surveyed. This was in agreement with flow cytometry results. In addition, the TEM sample preparation procedure involving repeated pelleting steps may have artificially enhanced the attachment observed in these images. Although the reason for this high heterogeneity in cell surface coverage by NPs is unclear, one hypothesis is that the NP surface chemistry that leads to aggregation also plays a vital role here. The uniform gap between the attached NPs and cell walls could be either the result of LPS that does not give significant TEM contrast or a double-layer (Debye length is ∼2 nm in our HEPES buffer).

*Shewanella* cells exposed to PAH–AuNPs were again lysed to a certain extent, shown in Fig. 6(c) but cells with AuNPs attached to the surface were not as ubiquitous as those observed on *Bacillus* surfaces. Second, instead of finding most of the nanoparticles on/near cell surfaces as observed on *Bacillus*, there are large clusters of AuNPs highly concentrated on cytoplasmic content spilled from lysed cells, indicated by the yellow dash-lined region in Fig. 6(f). Lastly, high magnification images reveal the presence of lipid bilayer-like structures localized with PAH–AuNPs (Fig. 6(f) and bottom inset in Fig. 6(c)), marked by red arrows. These structures are 4–6 nm in thickness, which is highly comparable to the expected thickness for a Gram-negative bacterial lipid bilayer. Interestingly, the top inset of Fig. 6(c) reveals an instance where the lipid bilayer with AuNP attached are still part of an intact cell wall membrane structure.

Images in Fig. 6(c) and (f) revealed a remarkable interaction between PAH–AuNPs with the Gram-negative bacteria. We hypothesize that through electrostatic attraction, PAH–AuNPs initially attach to the negative cell surfaces, leading to cell wall deformation and destruction. Zhao, *et.al.* have reported related studies observing cationic AuNPs attached to spilled nucleic acids from lysed *E. coli* cells.^13^ What is unique here is the cationic polyelectrolyte's strong interaction resulted in the attachment of AuNPs with fragmented membrane bilayers in the cellular debris. This observation also supports the hypothesis based on flow cytometry results that, at this concentration, PAH–AuNPs have access to the outer membrane bilayer upon micellization of LPS. The AuNP association with the lipid bilayer was not observed with *Bacillus* samples because of the differences in cell wall structures between the two strains.

## Conclusions

Bacteria are vital contributors to the environmental nutrient cycle and indicators for ecological health; thus, they are important single cell model organisms for assessing the impact of nanomaterials in the environment. Herein, we have systematically examined the toxicity of anionic MPA–AuNPs, cationic MPNH_2_–AuNPs, and cationic polyelectrolyte PAH–AuNPs on both *Shewanella* and *Bacillus*. Through a combination of *in situ* and *ex situ* methods of flow cytometry and electron microscopy, we have established a strong correlation between AuNP surface attachment on cells and bacterial viability. Concentration-dependent binding profiles of PAH–AuNPs on cell surfaces have revealed differences in the onset of binding between *Bacillus* and *Shewanella*. Electron micrographs from the same cell populations have revealed that although no NPs were internalized by either bacterial strain, both Gram-positive and Gram-negative membranes were severely damaged upon exposure to PAH–AuNP suspensions. Nanoparticles functionalized with cationic polyelectrolyte PAH, with the highest surface charge density of the nanoparticles employed, associated most significantly with bacterial surfaces and induced the greatest membrane damage and toxicity to both bacterial models. These results demonstrate the importance of a thorough understanding of the specific molecular interactions between AuNPs with well-tailored surface chemistries, the free ligands, and organism surfaces to guide the redesign of nanomaterials to avoid potential adverse effects in the environment. Alternatively, these results may also aid the design of novel antimicrobial drugs that target specific surface components of pathogens.
