# Supplementary material for: Impacts of gold nanoparticle charge and ligand type on surface binding and toxicity to Gram-negative and Gram-positive bacteria
Source: Chem Sci. 2015 Jun 16;6(9):5186–96. doi: 10.1039/c5sc00792e (PMC5669217; doi:10.1039/c5sc00792e)
Supplement: Supplementary file 2 [file SC-006-C5SC00792E-s002.pdf]

Electronic Supplementary Material (ESI) for Chemical Science.
This journal is © The Royal Society of Chemistry 2015

Impacts of Gold Nanoparticle Charge and Ligand Type on Surface Binding and Toxicity to Gram-Negative and Gram-Positive Bacteria

Z. Vivian Feng[a*], Ian L. Gunsolus[b], Tian A. Qiu[b], Katie R. Hurley[b], Lyle H. Nyberg[a], Hilena Frew[a], Kyle P. Johnson[b], Ariane M. Vartanian[c], Lisa M. Jacob[c], Samuel E. Lohse[c, 1], Marco D. Torelli[d], Robert J. Hamers[d], Catherine J. Murphy[c], and Christy L. Haynes[b*]

Supporting Information

Table of Contents:

- **Figure S1**. Representative transmission electron micrographs of MPA-, $MPNH_2$-, and PAH-AuNPs.
- **Figure S2**. Sample images from colony counting assays.
- **Figure S3**. Representative cytograms
- Calculation of PAH-ligand concentration on 5 μg/mL 4-nm PAH-AuNPs

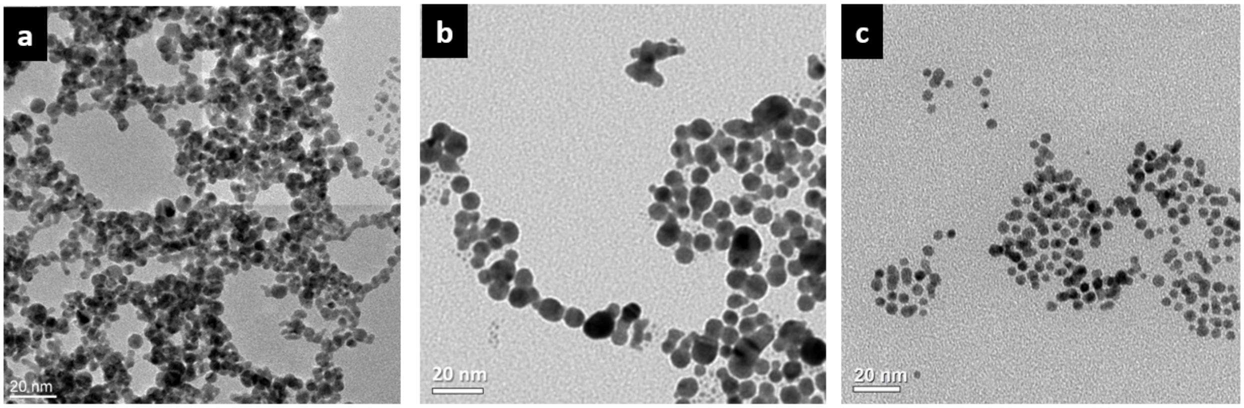

Figure S1. Representative transmission electron micrographs of (a) MPA-AuNPs, (b) $MPNH_2$-AuNPs, and (c) PAH-AuNPs.

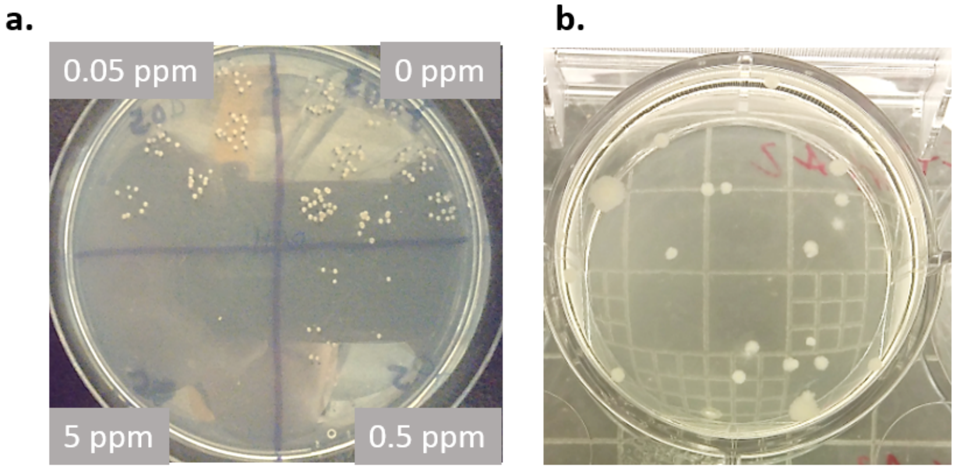

Figure S2. Representative results from plates used in colony counting methods: (a) drop plate for *Shewanella*, and (b) pour plate for *Bacillus*. The apparent variation in colony sizes in the *Bacillus* plate was due to the location of the colonies in the agar matrix, where smaller colonies were inside the matrix and the larger ones were on the surface.

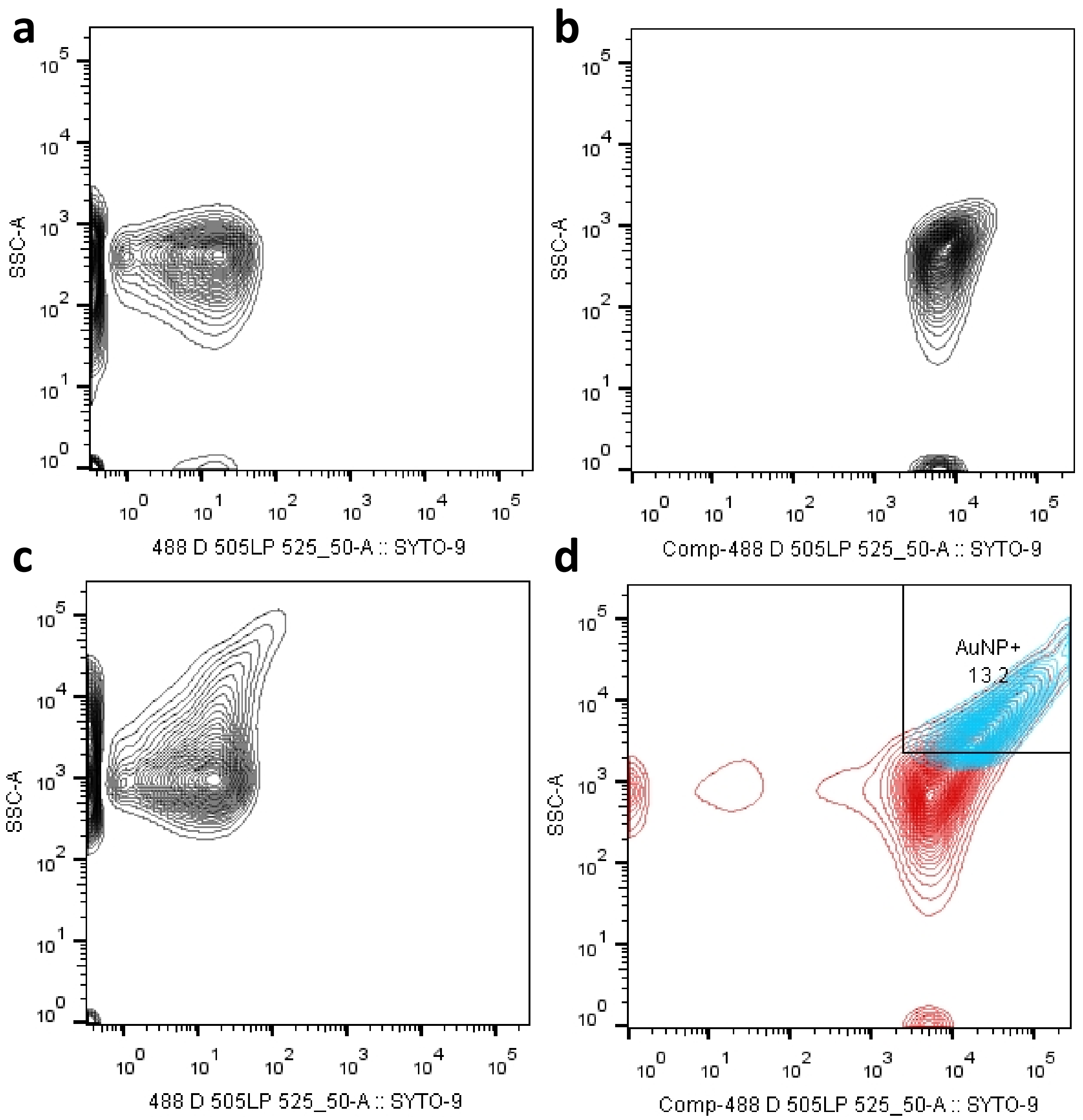

Figure S3. Sample cytograms of (a) unstained *Bacillus* cells, (b) SYTO 9-stained *Bacillus* cells, (c) AuNPs alone, and (d) SYTO 9-stained *Bacillus* with 5 μg/mL MPNH$_2$-AuNPs. Both axes are in arbitrary units corresponding to detector signals (vertical: side scattering detector at $\lambda = 488 \pm 10$ nm); horizontal: fluorescence detector channel at $\lambda = 510 \pm 10$ nm).

Determination of PAH-ligand concentration in 5 μg/mL 4-nm PAH-AuNPs

$$AuNPdiameter = 4nm$$

$$Volume_{AuNP} = \frac{4}{3}\pi(\frac{4}{2})^3 = 33.5nm^3 = 3.35\times10^{-20}cm^3$$

$$Density_{Au} = 19.3g/cm^3$$

$$Mass_{Au} = (3.35\times10^{-20}cm^3)\times(19.3g/cm^3) = 6.47\times10^{-19}g$$

$$\#AuNP/L = \frac{(5mg/L)\times(0.001g/mg)}{6.47\times10^{-19}g} = 7.73\times10^{15}$$

$$M_w ofPAH = 15000g/mol$$

$$\#PAH/\#AuNP = 4$$

$$[PAH](\mu g/mL) = \frac{(15000g/mol)\times4\times(7.73\times10^{15}/L)\times(1000mg/g)}{6.02\times10^{23}/mol} = 0.77mg/L = 0.77\mu g/mL$$
